# HumMeth27QCReport: an R package for quality control and primary analysis of Illumina Infinium methylation data

**DOI:** 10.1186/1756-0500-4-546

**Published:** 2011-12-19

**Authors:** Francesco M Mancuso, Magda Montfort, Anna Carreras, Andreu Alibés, Guglielmo Roma

**Affiliations:** 1 Bioinformatics Unit, Centre for Genomic Regulation (CRG) and UPF, Dr. Aiguader 88, 08003 Barcelona, Spain; 2 Genotyping Unit-Centro Nacional de Genotipado (CeGen), Centre for Genomic Regulation (CRG) and UPF, Dr. Aiguader 88, 08003 Barcelona, Spain; 3 Genes and Disease Programme, Centre for Genomic Regulation (CRG) and UPF, Dr. Aiguader 88, 08003 Barcelona, Spain

## Abstract

**Background:**

The study of the human DNA methylome has gained particular interest in the last few years. Researchers can nowadays investigate the potential role of DNA methylation in common disorders by taking advantage of new high-throughput technologies. Among these, Illumina Infinium assays can interrogate the methylation levels of hundreds of thousands of CpG sites, offering an ideal solution for genome-wide methylation profiling. However, like for other high-throughput technologies, the main bottleneck remains at the stage of data analysis rather than data production.

**Findings:**

We have developed *HumMeth27QCReport*, an R package devoted to researchers wanting to quickly analyse their Illumina Infinium methylation arrays. This package automates quality control steps by generating a report including sample-independent and sample-dependent quality plots, and performs primary analysis of raw methylation calls by computing data normalization, statistics, and sample similarities. This package is available at CRAN repository, and can be integrated in any Galaxy instance through the implementation of ad-hoc scripts accessible at Galaxy Tool Shed.

**Conclusions:**

Our package provides users of the Illumina Infinium Methylation assays with a simplified, automated, open-source quality control and primary analysis of their methylation data. Moreover, to enhance its use by experimental researchers, the tool is being distributed along with the scripts necessary for its implementation in the Galaxy workbench. Finally, although it was originally developed for HumanMethylation27, we proved its compatibility with data generated with the HumanMethylation450 Bead Chip.

## Findings

DNA methylation is an epigenetic mechanism that in vertebrates occurs most frequently at cytosines followed by guanines (CpG). This modification regulates gene expression and can be inherited through cell division, thus being essential for preserving tissue identities and guiding normal cellular development [1]. Recent studies have also indicated that modulation of DNA methylation occurs during lineage-specific differentiation. For instance, methylation and transcriptional changes accompany myeloid versus lymphoid fate decisions [2]. However, alterations in DNA methylation at specific regulatory sites have been linked to common diseases such as cancer, diabetes, multiple sclerosis, schizophrenia, and other neurodegenerative disorders [3-5].

As investigating the human DNA methylome has gained more and more interest in the last few years, several methods have been developed to detect cytosine methylation on a genomic scale. Among these, the Illumina Infinium Methylation Assay has proven reliable in genotyping labs worldwide. This hybridization-based technique offers quantitative methylation measurements at the single-CpG-site level and provides as accurate results as sequencing-based methylation assays (e.g. MethylCap-seq, MeDIP-seq, RRBS) [6].

More specifically, two Illumina platforms are available for high-throughput DNA methylation analysis so far: Golden Gate Veracode and Infinium. The Veracode Golden Gate Methylation represents an ideal platform for custom studies or biomarker validation, targeting 96 or 384 custom CpG sites per sample. For Infinium platform, two different Bead Chips are on the market: the HumanMethylation27 and the new HumanMethylation450 Bead Chips. The first enables scientists to interrogate 27,578 highly informative CpG sites located within the genomic regions upstream of 14,475 consensus coding sequence (CCDS) annotated at the NCBI database. Instead, the new HumanMethylation450 Bead Chip provides scientists with methylation levels of >450,000 CpG sites per sample, including coverage of all designable RefSeq genes (along with promoter, 5', and 3' regions, without bias against those lacking islands of CpG sites), CpG sites outside of CpG islands, non-CpG methylated sites identified in human stem cells, differentially methylated sites identified in tumor versus normal in multiple forms of cancer and across several tissue types, CpG islands outside of coding regions, microRNA promoter regions, and disease-associated regions identified through genome-wide association studies.

Like for other technologies, quality control is an important step in the analysis of methylation array data [7]. Indeed, arrays with poor quality can significantly affect subsequent analysis and consequently invalidate their interpretation. But, while several R packages can assist scientists with quality control analysis of other data types (i.e. microarray [8-10], RNA-seq [11], target enrichment experiments [12]), only four are tailored to support DNA methylation research and, in particular, only two specifics for Illumina data. Of these, charm [13] implements analysis tools for DNA methylation data generated using Nimblegen microarrays and the McrBC protocol; it finds differentially methylated regions between samples, calculates percentage methylation estimates and includes array quality assessment tools. MassArray [14] is designed for the import, quality control, analysis, and visualization of methylation data generated using Sequenom's MassArray platform. Specific for Illumina, *methylumi *[15] facilitates data manipulation with methods for quality control, normalization (only for Illumina Golden Gate), and plotting; while *lumi *[16] handles Infinium data providing functions for quality and colour balance assessment, colour balance adjustment, background adjustment, normalization, and modelling of the methylation status. While these open source packages require knowledge and live interaction with the R programming environment, non-computational scientists could instead carry out the analysis with the Illumina Genome Studio software. However, since not all researchers may have access to this commercial software, we believe that a fully automated, open source and unique function-based tool would facilitate the analysis of methylation data even for wet-lab researchers.

**Figure 1:**
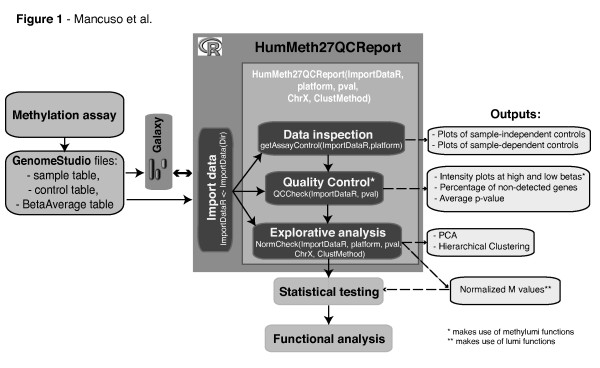
**HumMeth27QCReport pipeline**. Standard analysis workflow of Illumina Methylation Assay data. Steps facilitated by H*umMeth27QCReport *are included in dark gray box.

Here, we developed *HumMeth27QCReport*: an R package to automate quality control and analysis of Illumina Infinium Methylation Arrays. This software is made of a unique function that performs four main steps (Figure 1), some of which based on already existing functionalities from *lumi *and *methylumi*. First, *HumMeth27QCReport *imports methylation files directly as they are exported from *Genome Studio *without any further manipulation. An optional file can be provided to eventually discard some samples from subsequent analysis. Second, *HumMeth27QCReport *generates ad-hoc quality plots to monitor in an easy and fast way sample-dependent and sample-independent QC parameters. These plots, designed with wet-lab researchers following the Illumina guidelines, are automatically exported in printable PDF format. Third, *HumMeth27QCReport *makes use of normalization method functionality of *lumi *package, as well as exports normalized methylation calls and basic statistics in text format that can be easily imported into any statistical package or spreadsheet program for further analyses. Fourth, *HumMeth27QCReport *computes Principal Component Analysis (PCA) and hierarchical clustering to assess overall sample similarities on normalized DNA methylation M-values. Importantly, we show that this software, originally developed for HumanMethylation27, is compatible with data produced with the new HumanMethylation450 Bead Chip. Finally, the package is available at CRAN repository, and, in order to encourage its use by non-computational researchers, ad-hoc scripts for its implementation in the Galaxy workbench [17,18] are provided at the Galaxy Tool Shed [19].

### DNA methylation samples and analysis

Three control samples with different global methylation levels were used to test the new Infinium Human Methylation450 Bead Chip technology. In the assay, 750 ng of methylated Jurkat gDNA (New England Biolabs, UK), Jurkat gDNA (New England Biolabs, UK) and unmethylated gDNA obtained by whole-genome amplification (GenomiPhi V2 DNA Amplification Kit, GE Healthcare, UK) were treated with sodium bisulfite using EZ DNA Methylation-Gold (Zymo Research, CA, USA) and EpiTect Bisulfite Kits (QiaGen) following the manufacturer standard protocol. Two different bisulfite conversions were performed per each sample using both kits. Therefore, 12 bisulfite-converted DNA samples were obtained at the end of this process. In addition, as example of sample of poor quality, we added a real sample coming from human amygdala (AMYG) brain area of a sporadic Parkinson case (Table 1). For the latter, genomic DNA was isolated from the tissue samples using the Master Pure DNA purification kit (Epicentre Biotechnologies), following manufacturer's specifications, and then bisulfite treated using the EZ-96 Methylation Kit (Zymo Research).

**Table 1:** Samples used for the analysis.

ID	Sample Name
1	JUR KIT2_BIO1

2	JUR KIT2_BIO2

3	JUR KIT1_BIO1

4	JUR KIT1_BIO2

5	JUR MET KIT2_BIO1

6	JUR MET KIT2_BIO2

7	JUR MET KIT1_BIO1

8	JUR MET KIT1_BIO2

9	UNMET KIT2_BIO1

10	UNMET KIT2_BIO2

11	UNMET KIT1_BIO1

12	UNMET KIT1_BIO2

13	PD3 AMYG

KIT1 = EZ DNA Methylation-Gold; KIT2 = EpiTect Bisulfite Kits

Infinium Human *Methylation450 *Bead Chip was hybridized with the treated samples according to the standard protocol provided by Illumina. The fluorescently stained chip was imaged by the Illumina iScan Reader. Genome Studio Methylation Module was used to analyze intensities to assign site-specific DNA methylation β-values to each CpG site. *HumMeth27QCReport *was used to analyse raw files exported from Genome Studio software using default options: significance level p-value cutoff of 0.05, option to discard CpGs located on chromosome X set to false, and "Euclidean" distance for the clustering analysis.

### Implementation

*HumMeth27QCReport *is an extension package for the programming language and statistical environment R [20] (currently implemented for the version 2.12.1). The package has been developed to analyze the Illumina Infinium HumanMethylation27 Bead Chip, but it is already compatible with the new HumanMethylation450 panel. *HumMeth27QCReport *integrates functions from other packages like *methylumi *for data import and dye bias check, *lumi *for normalization, *amap *and *Hmisc *for PCA and clustering. It requires the R packages *gplots*, *plotrix*, *WriteXLS*, *tcltk *for graphics and data export steps, and the installation of Perl programming language to run the *WriteXLS *package.

An interface has also been designed for integrating *HumMeth27QCReport *into the Galaxy workbench and it is available through the Galaxy Tool Shed for anyone to add it to its own Galaxy instance.

### Analysis Workflow

The package workflow is summarized in Figure 1. *HumMeth27QCReport *requires as input three tab-delimited text files exported from the Genome Studio Methylation Module ("Sample table", "Control probe profile", and "Average Beta table") and reads into R using the basic function read. table(). After data import, the package first computes basic statistics, and then generates quality plots to monitor the Illumina Infinium sample-independent and sample-dependent internal quality controls. More in detail, sample-independent controls evaluate specific experimental steps and include:

• staining controls, which are used to examine the efficiency of the staining step;

• extension controls, which test the extension efficiency of A, T, C, and G nucleotides from a hairpin probe;

• target removal controls, which test the efficiency of the stripping step after the extension reaction. All target removal controls should result in low signal compared to the hybridization controls, indicating that the targets are removed efficiently after extension;

• hybridization controls, which test the overall performance of the entire assay using synthetic targets present at different concentrations.

Instead, sample-dependent controls evaluate performance across samples and include:

• bisulfite conversion controls, which assess the efficiency of bisulfite conversion of the genomic DNA;

• specificity controls, which monitor potential non-specific primer extension. Perfectly Matched (PM) controls correspond to A/T perfect match and should give high signal, instead Mismatched (MM) controls correspond to G/T mismatch and should give low signal;

• negative controls. The negative control probes are randomly permutated sequences that should not hybridize to the DNA template. The mean signal of these probes defines the system background;

• non-Polymorphic (NP) controls, which test the overall performance of the assay, from amplification to detection, by querying a particular base in a non-polymorphic region of the bisulfite genome.

For each of the above-listed internal controls, *HumMeth27QCReport *generates plots to represent the percentage of background on signal and the percentage of CpGs not detectable at two different p-value cut-offs (0.01 and 0.05), as well as evaluates the average detection p-value of each sample. Low performance samples can be automatically removed from further analyses based on a user-defined cut-off (default p-value cut-off is 0.05).

Then, the package integrates *methylumi *and *lumi *functionalities to monitor and correct the dye-bias that is a common problem in two-colour microarray experiments [20,21]. Firstly, it applies *methylumi *to plot intensities distribution at different cut-offs to easily monitor eventual dye bias present in the arrays; secondly, it performs quantile normalization provided by *lumi *[16] to correct the eventual dye-bias. The assumption of quantile normalization is that the intensity distribution of the pooled methylated and unmethylated probes are similar for different samples. Because the total amount of CpG methylation can be significantly different from sample to sample in different conditions, a direct application of the normalization methods designed for expression microarray to the methylation data is inappropriate. To avoid this problem, our tool inherits the *lumiMethyN *function from the *lumi *package for probe level normalization (i.e., normalize the intensities of methylated and unmethylated probes instead of the summarized methylation levels).

Normalized M-values, defined in [16] and [22], are then used to compute Principal Component Analysis (PCA) and hierarchical clustering in order to assess sample similarities. We decided to use M-values instead of Beta-value because the first are more statistically valid for the differential analysis of methylation levels. As described in Du et al., M-values are calculated as the log2 ratio of the intensities of methylated probe versus unmethylated probe. An M-value close to 0 indicates a similar intensity between the methylated and unmethylated probes, which means that the CpG site is about half-methylated. Positive M-values is associated to a hyper-methylation while negative M-values mean the opposite.

The following basic statistics are exported into an excel file, to give a more accurate view of the quality of the dataset:

• summary values of the internal controls;

• summary of percentage of not-detected genes and average p-value for each sample;

• list of not-detected CpGs with p-value greater than 0.01 in more than 5% of the samples;

• list of not-detected CpGs with p-value greater than 0.05 in more than 5% of the samples.

Finally, the M-values are automatically exported into a text file so that they can be employed in following analysis such as identification of differentially methylated genes using the R Bioconductor package *limma *[23].

### QC analysis of HumanMethylation450 Bead Chip arrays

In order to test the compatibility of our package with the new HumanMethylation450 Bead Chip, we performed the analysis of 12 control samples divided in 3 groups differently methylated: Jurkat (JUR), artificially methylated Jurkat (JUR MET), and unmethylated (UNMET). Samples in each group were treated with two different sodium bisulfite kits, QiaGen or EZ Gold. In addition, we included a sample with poor quality in the analysis (Table 1).

**Figure 2:**
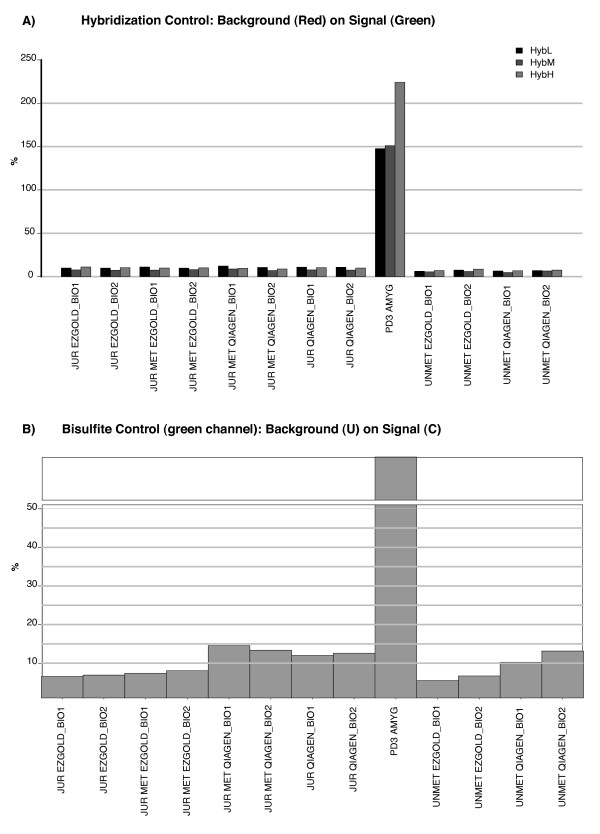
**Internal controls**. Examples of sample-independent (A) and sample-dependent (B) plots generated by the *HumMeth27QCReport *package. **a**. *Hybridization control*, to check synthetic targets at low, medium, and high concentration level. Low percentage for each level is an indication of good performance; **b**. *bisulfite control *on green channel, to monitor the efficiency of genomic-DNA bisulfite conversion using the Infinium I assay design. Low percentage is an indication of good performance, whereas if the percentage is high we consider that the sample has lower performance. A detailed explanation of each plot is available at the *HumMeth27QCReport *homepage (http://biocore.crg.es/wiki/HumMeth27QCReport). JUR: Jurkat DNA; JUR MET: methylated Jurkat DNA; UNMET: unmethylated DNA; EZGOLD: EZ DNA Methylation-Gold Kit (Zymo Research, CA, USA); QIAGEN: EpiTect Bisulfite Kit (QiaGen); BIO1: biological replicate 1; BIO2: biological replicate 2; PD3 Amyg: example of sample of poor quality.

The automatic report generated by our package indicates that all control samples had similar performance, since they all had comparable intensity levels for all internal controls. The overall low ratios between background and signal showed a high efficiency for each sample-dependent and -independent parameter. On the contrary, the sample with poor quality presents very high ratios (or low absolute values). In particular, hybridization control and bisulfite conversion, monitored in the green channel, turned out to be particularly significant (Figure 2). Overall, the control samples had an average detection p-value below the cut-off set for this analysis (*p *< 0.05) and their biological replicates showed a very high reproducibility (Pearson correlation r > 0.95); instead the poor quality sample had a p-value ten times higher than the cut-off and more than 90% of non detected genes. Finally, PCA and hierarchical clustering methods, computed only on those samples that passed the p-value threshold, suggested that the main differences among them might derive from their methylation levels (JUR, JUR MET, UNMET) and not from the treatment (QiaGen, EZ Gold). A full QC analysis report is available in Additional file 1.

## Conclusions

*HumMeth27QCReport *is an R package designed with wet lab researchers for automating the quality control and primary analysis of Illumina Infinium Methylation Assays. The novelty of our software is the automatic generation of ad-hoc quality plots that allow an easy monitor of sample-dependent and sample-independent QC parameters, as suggested by the Illumina Infinium guidelines. These plots represent a comprehensive tool for visual inspection of methylation datasets. Moreover, our software exports DNA methylation calls and basic statistics into text files that can be easily imported into other statistical software for further analysis, as well as computes sample similarities using unsupervised approaches, like PCA and hierarchical clustering. The software, compatible with the new HumanMethylation450 Bead Chip, is available at the CRAN repository. To enhance its use by non-computational researchers, we developed ad-hoc scripts for its implementation in the Galaxy workbench and made them available at the Galaxy Tool Shed.

## Availability and requirements

**Project name: **HumMeth27QCReport

**Project homepage: **http://biocore.crg.cat/wiki/HumMeth27QCReport;http://cran.r-project.org/package=HumMeth27QCReport

**Operating system(s): **Platform independent

**Programming language: **R

**Dependecies: **R (> = 2.13.0), methylumi, lumi, IlluminaHumanMethylation27k.db, IlluminaHumanMethylation450k.db, amap, Hmisc, gplots, plotrix, WriteXLS, tcltk

**Other requirements: **Perl

**License: **GNU GPLv2

## Availability of supporting data

The data set supporting the results of this article and a further example data set are available at the tool homepage: http://biocore.crg.cat/wiki/HumMeth27QCReport

## Competing interests

The authors declare that they have no competing interests.

## Authors' contributions

FMM and GR developed the R package, and wrote the manuscript. AC and MM participated in its design, particularly in the discussion of the QC plots to be implemented, and in the preparation of the manuscript. AA developed the Galaxy implementation of the package. All authors read and approved the final manuscript.

## Supplementary Material

Additional file 1 **Full QC report PDF file containing all the figures produced by HumMeth27QCReport during the QC analysis**. Click here for file
