# Supplementary material for: HumMeth27QCReport: an R package for quality control and primary analysis of Illumina Infinium methylation data
Source: BMC Res Notes. 2011 Dec 19;4:546. doi: 10.1186/1756-0500-4-546 (PMC3285701; doi:10.1186/1756-0500-4-546)

Supplementary file 1 – Full QC report

Here we report an example of automatic QC report generated with *HumMeth27QCReport* on a DNA methylation experiments conducted with the Illumina Infinium Human Methylation450 BeadChip. In the experiment, 3 different samples were bisulfite converted using 2 different kits. Each bisulfite conversion was performed twice. Finally, 12 control samples were hybridized at the end of this process (Table I). Each HumanMethylation450 BeadChip consists of 12 arrays, so in our explorative analysis all bisultife conversions were hybridized on the sample BeadChip. Moreover, to give a better idea of how a bad sample should look like, we included in the analysis a poor quality sample: an amygdala (AMYG) brain tissue that was processed in an independent assay.

- ***Barplots of Illumina Internal Control (page 3-17).*** Percentage of background on signal was represented in barplots for most of the internal controls. Samples with low performance usually fail for one or more plots, where higher percentage of background versus signal is observed versus samples with good performance. A rapid evaluation of the plots allows you to identify if the low performance can be related with DNA quality (Sample Dependent-Controls) or assay performance (Sample-Independent Controls). In our particular example all controls samples have similar performance among them, so all have comparable intensity levels for all internal controls; on the contrary the bad sample presents a very high ratios, synonym of very low quality.
- ***Intensity at high and low betas (page 18).*** This is a graphical example of this dye bias of non-normalized intensities. For each of the Cy3 and Cy5 channels, a cutoff in beta values is used to calculate which Cy3 and Cy5 values should be plotted at high-methylation (cutoff of 0.8) and low-methylation status (cutoff of 0.2). When plotted in this way, any offset between Cy3 and Cy5 way likely represents dye bias and will lead to biases in the estimation of beta. In our example only twelve plots were produced because the bad sample has no values at the high cutoff.
- ***Percentage of non detected-genes (page 19)*** at two different p-value cut-offs (0.01 and 0.05). This plot gives us an immediate overview of the global CpG coverage. In our example all control samples achieved >99% CpG site call rate at both significance levels (so the bars are closed to 0 in the graphical representation) meanwhile bad sample have less than 10% of detected CpGs.
- ***Average p−value of each sample (page 20).*** The barplots show the average pvalue for each sample; the red dotted line is the threshold defined by the user to select the samples for the following analysis. In our example, the threshold was defined at 0.05 significance level.
- ***Principal Component Analysis (page 21)*** on filtered and normalized data only for those samples that passed the average p-value threshold. In our experimental analysis samples were grouped in three clusters: Jurkat DNA, Jurkat methylated DNA and unmethylated DNA.
- ***Hierarchical Clustering (page 22)*** on filtered and normalized data only for those samples that passed the average p-value threshold. As shown in the PCA, the main differences among samples derive from the methylation profiles of the samples.

Sample List

Index	SampleID	Index	SampleID
1	JUR QIAGEN_BIO1	8	JUR MET EZGOLD_BIO2
2	JUR QIAGEN_BIO2	9	UNMET QIAGEN_BIO1
3	JUR EZGOLD_BIO1	10	UNMET QIAGEN_BIO2
4	JUR EZGOLD_BIO2	11	UNMET EZGOLD_BIO1
5	JUR MET QIAGEN_BIO1	12	UNMET EZGOLD_BIO2
6	JUR MET QIAGEN_BIO2	13	PD3 AMYG
7	JUR MET EZGOLD_BIO1		

Staining DNP Control: Background (BGND) on Signal (MED)

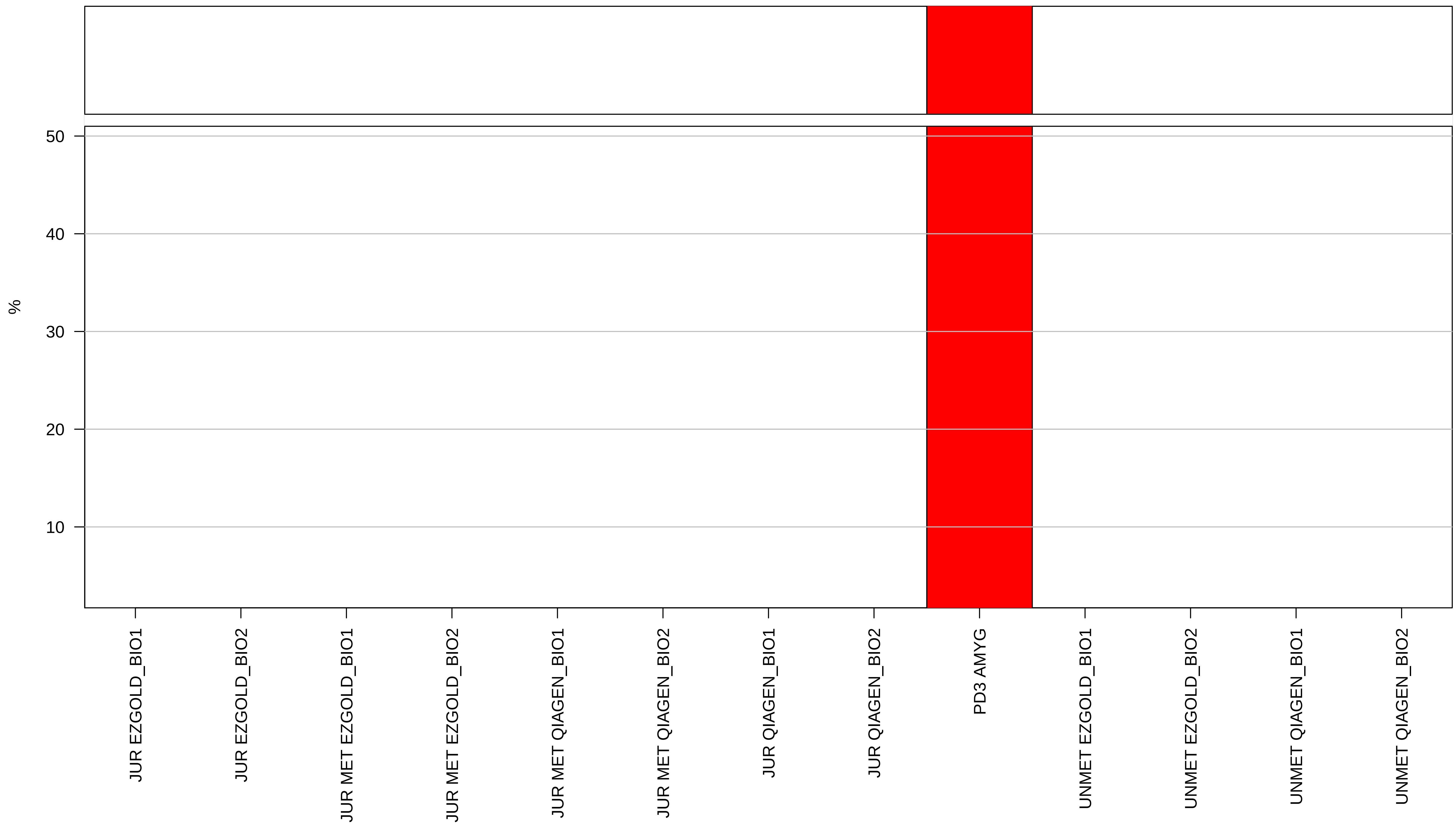

Staining Biotin Control: Background (BGND) on Signal (MED)

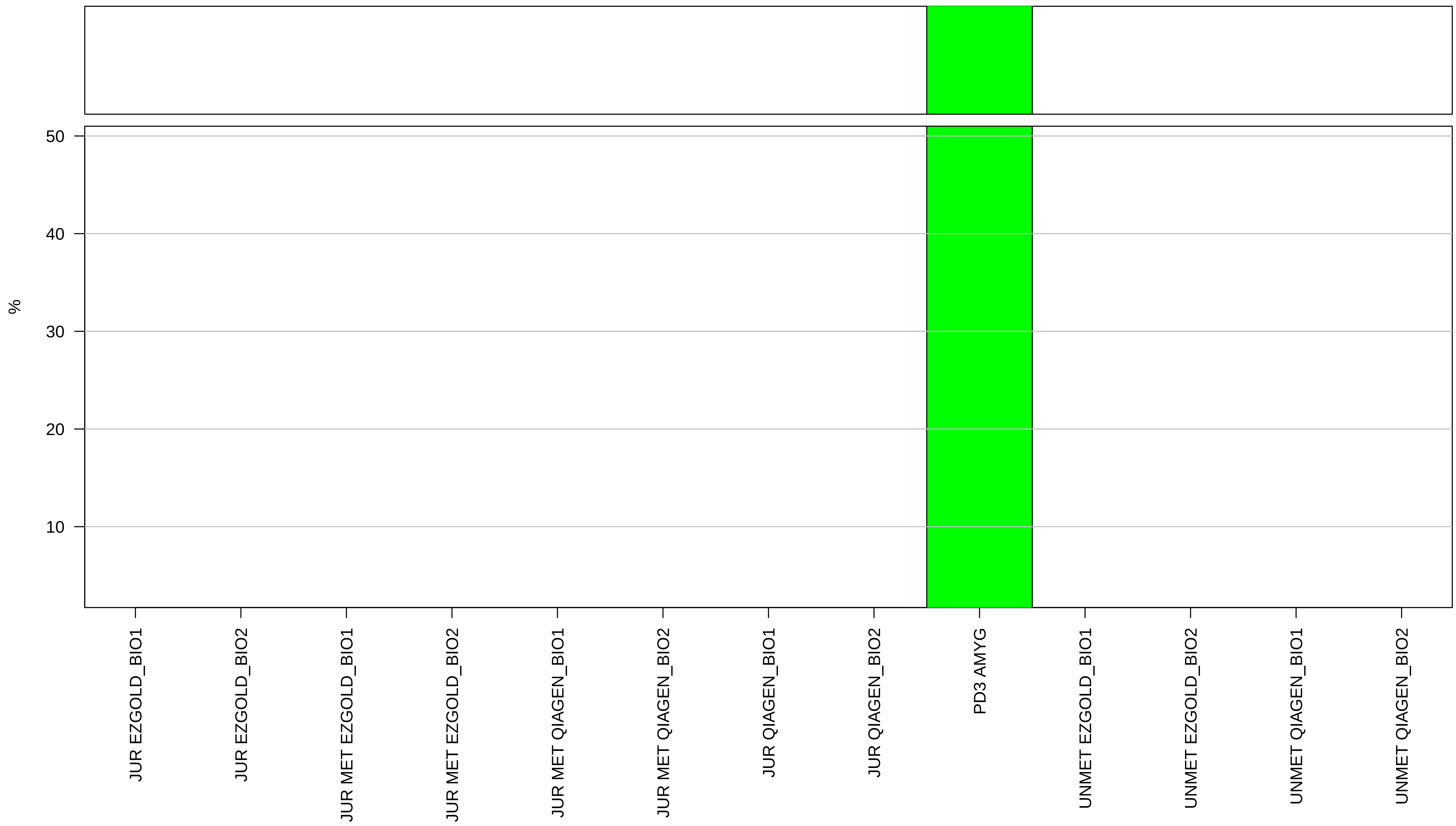

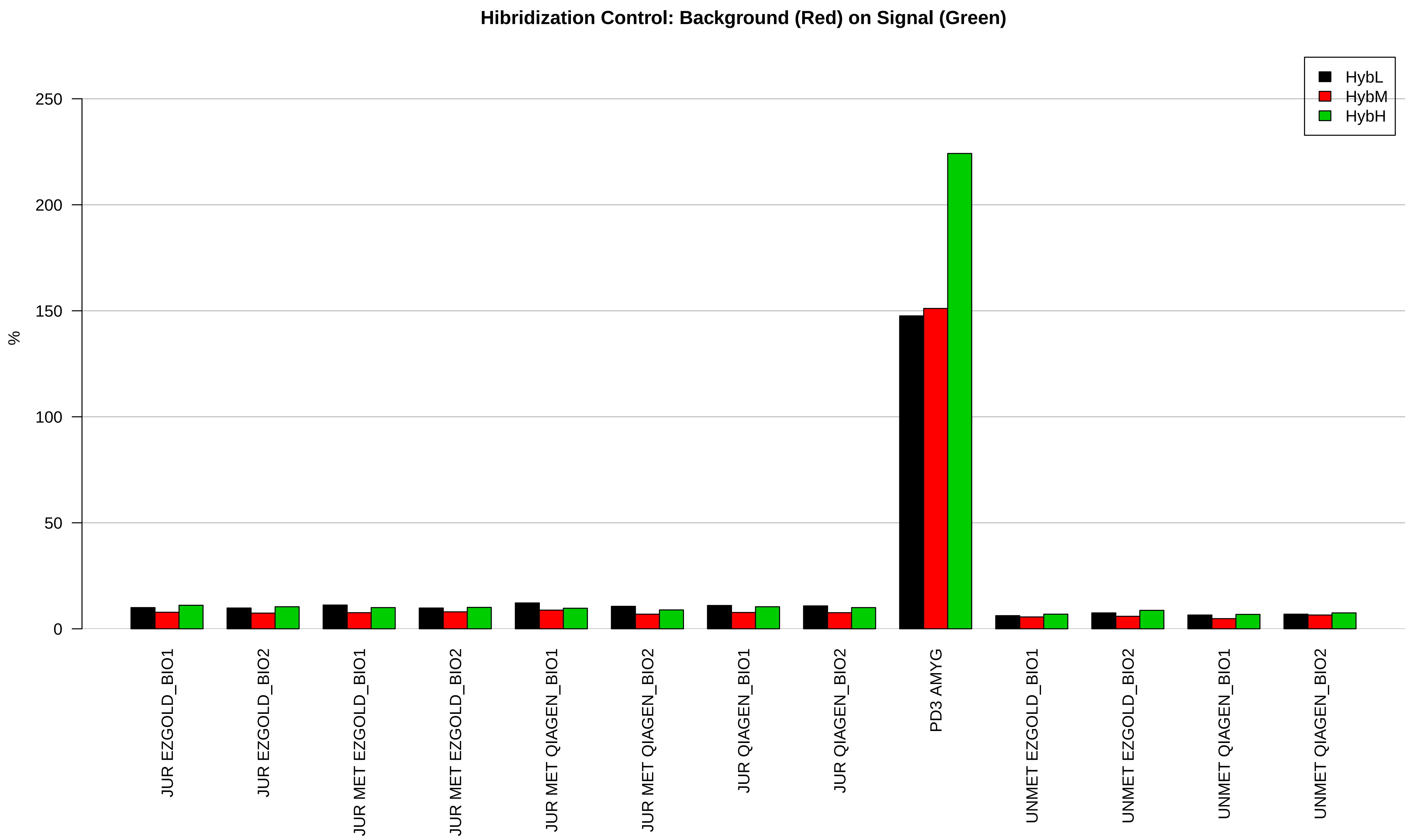

Hibridization Control: Background (Red) on Signal (Green)
HybL
HybM
HybH
%
250
200
150
100
50
0
JUR EZGOLD_BIO1
JUR EZGOLD_BIO2
JUR MET EZGOLD_BIO1
JUR MET EZGOLD_BIO2
JUR MET QIAGEN_BIO1
JUR MET QIAGEN_BIO2
JUR QIAGEN_BIO1
JUR QIAGEN_BIO2
PD3 AMYG
UNMET EZGOLD_BIO1
UNMET EZGOLD_BIO2
UNMET QIAGEN_BIO1
UNMET QIAGEN_BIO2

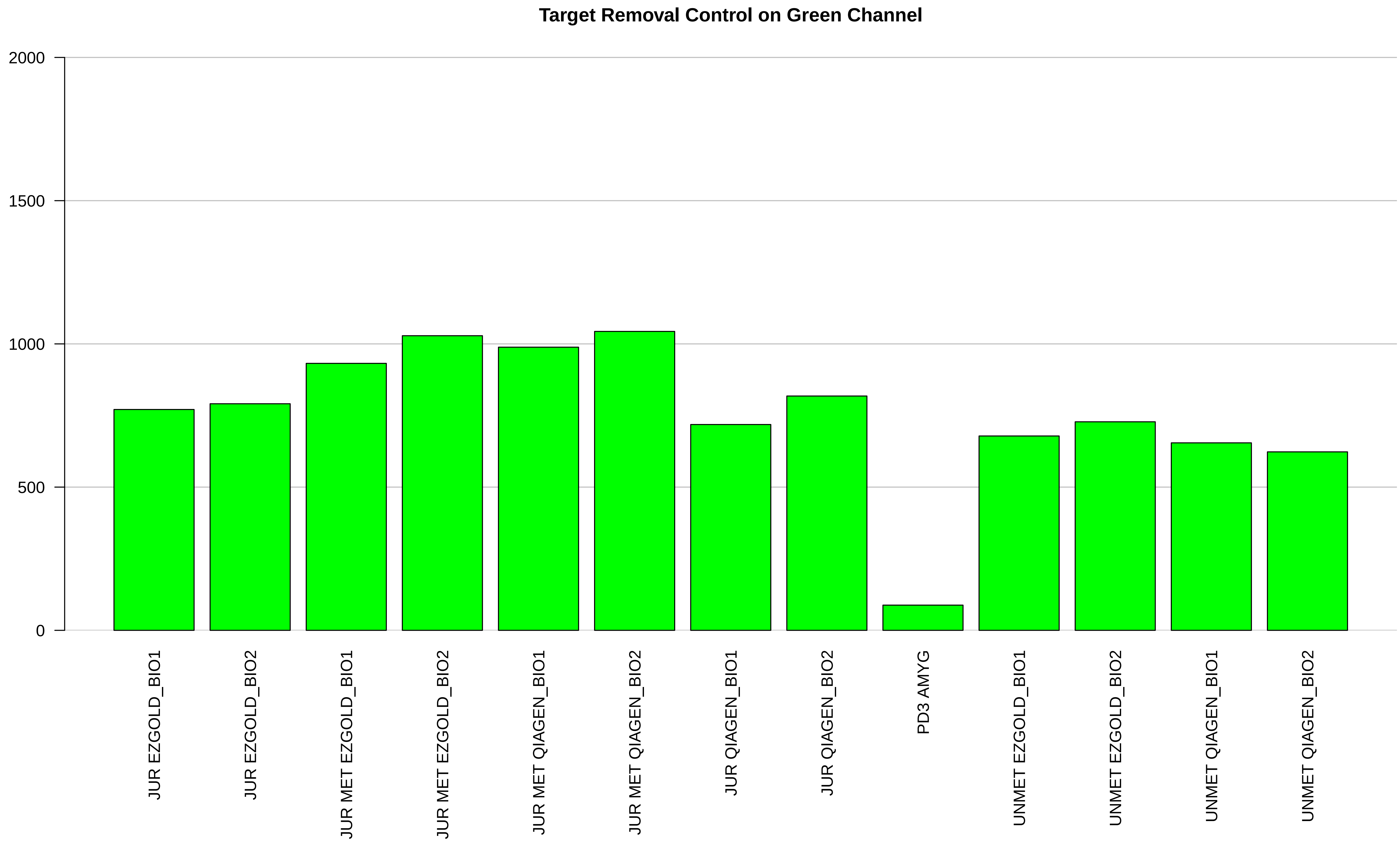

Target Removal Control on Green Channel
2000
1500
1000
500
0
JUR EZGOLD_BIO1
JUR EZGOLD_BIO2
JUR MET EZGOLD_BIO1
JUR MET EZGOLD_BIO2
JUR MET QIAGEN_BIO1
JUR MET QIAGEN_BIO2
JUR QIAGEN_BIO1
JUR QIAGEN_BIO2
PD3 AMYG
UNMET EZGOLD_BIO1
UNMET EZGOLD_BIO2
UNMET QIAGEN_BIO1
UNMET QIAGEN_BIO2

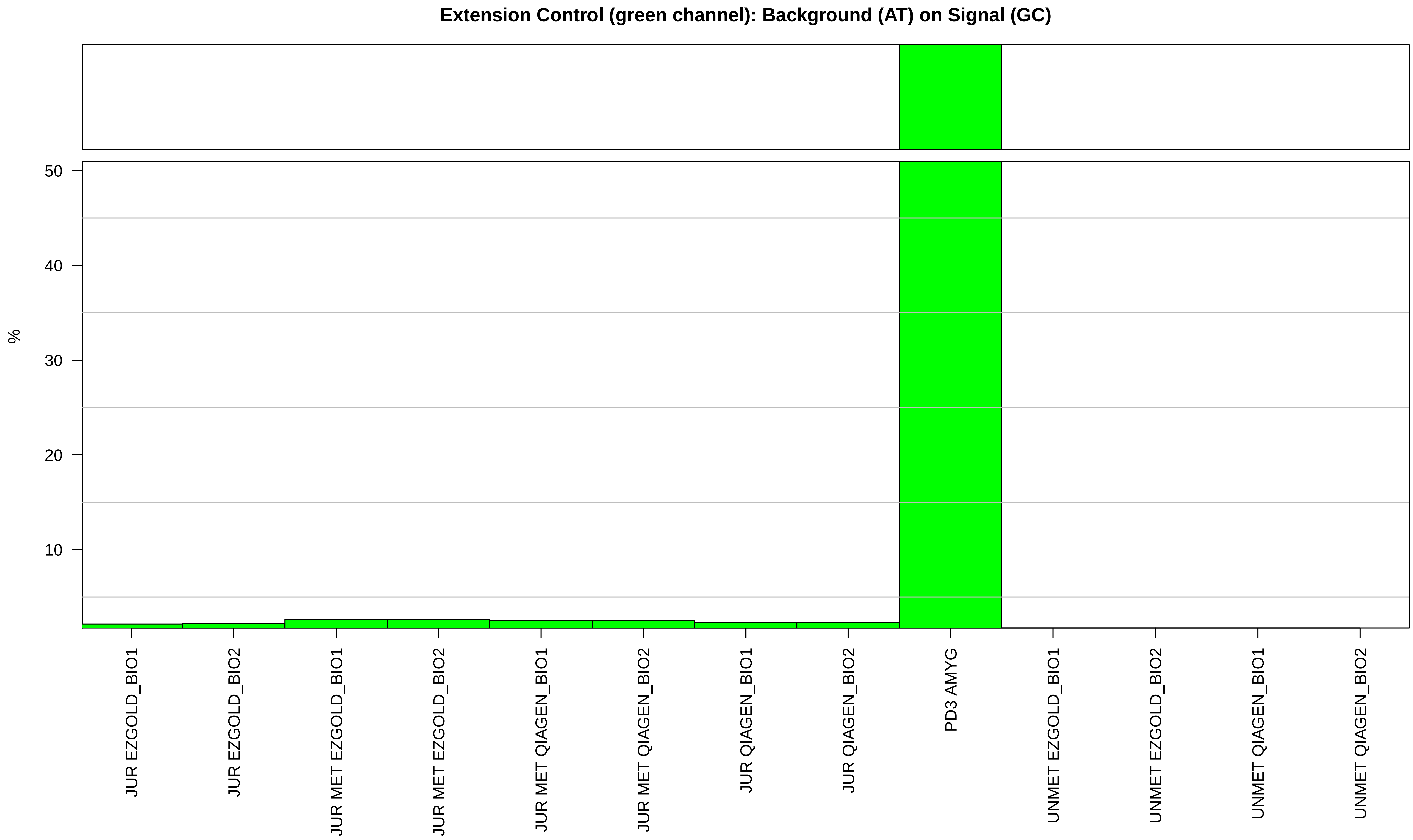

Extension Control (green channel): Background (AT) on Signal (GC)
%
50
40
30
20
10
JUR EZGOLD_BIO1
JUR EZGOLD_BIO2
JUR MET EZGOLD_BIO1
JUR MET EZGOLD_BIO2
JUR MET QIAGEN_BIO1
JUR MET QIAGEN_BIO2
JUR QIAGEN_BIO1
JUR QIAGEN_BIO2
PD3 AMYG
UNMET EZGOLD_BIO1
UNMET EZGOLD_BIO2
UNMET QIAGEN_BIO1
UNMET QIAGEN_BIO2

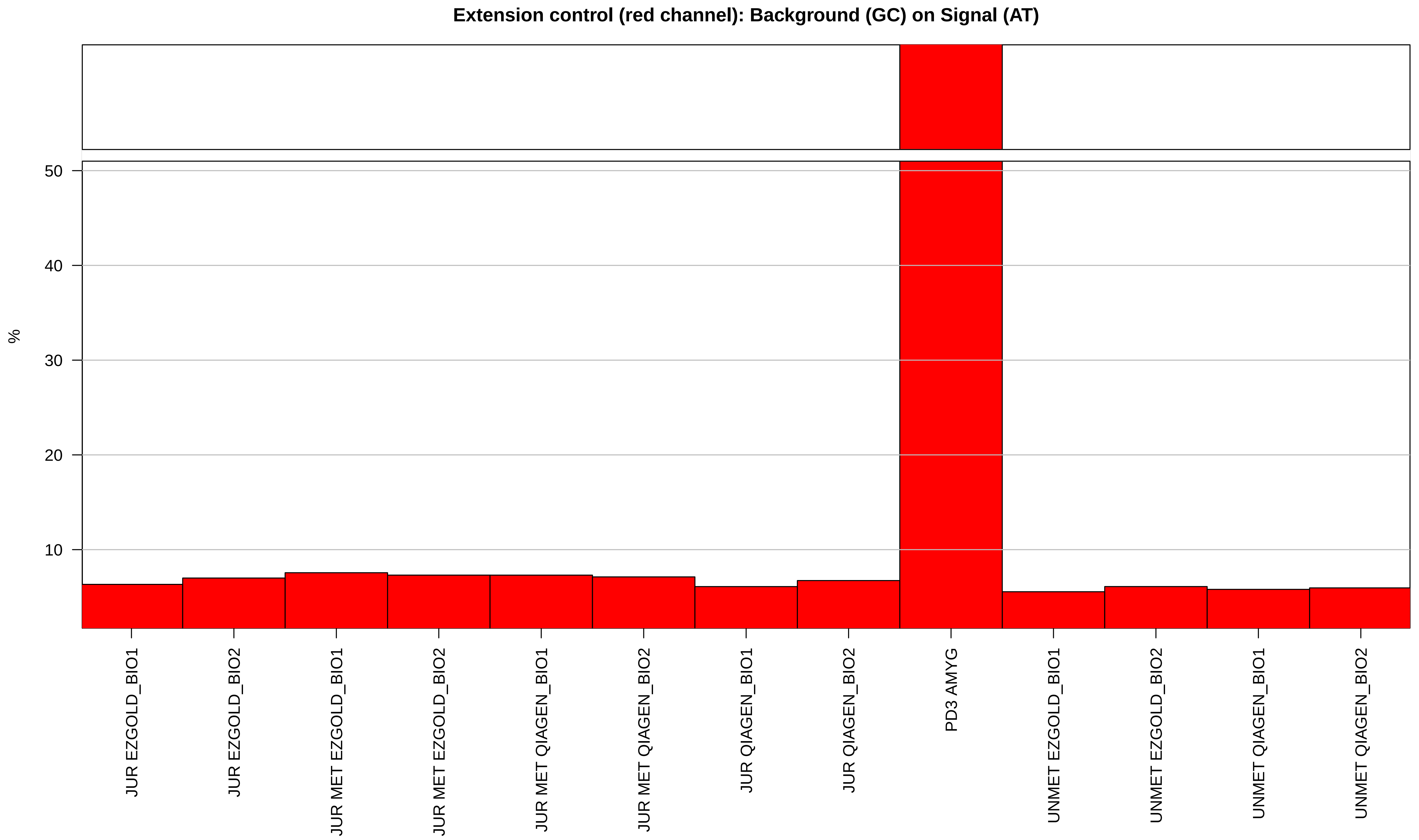
Extension control (red channel): Background (GC) on Signal (AT)
%
50
40
30
20
10
JUR EZGOLD_BIO1
JUR EZGOLD_BIO2
JUR MET EZGOLD_BIO1
JUR MET EZGOLD_BIO2
JUR MET QIAGEN_BIO1
JUR MET QIAGEN_BIO2
JUR QIAGEN_BIO1
JUR QIAGEN_BIO2
PD3 AMYG
UNMET EZGOLD_BIO1
UNMET EZGOLD_BIO2
UNMET QIAGEN_BIO1
UNMET QIAGEN_BIO2

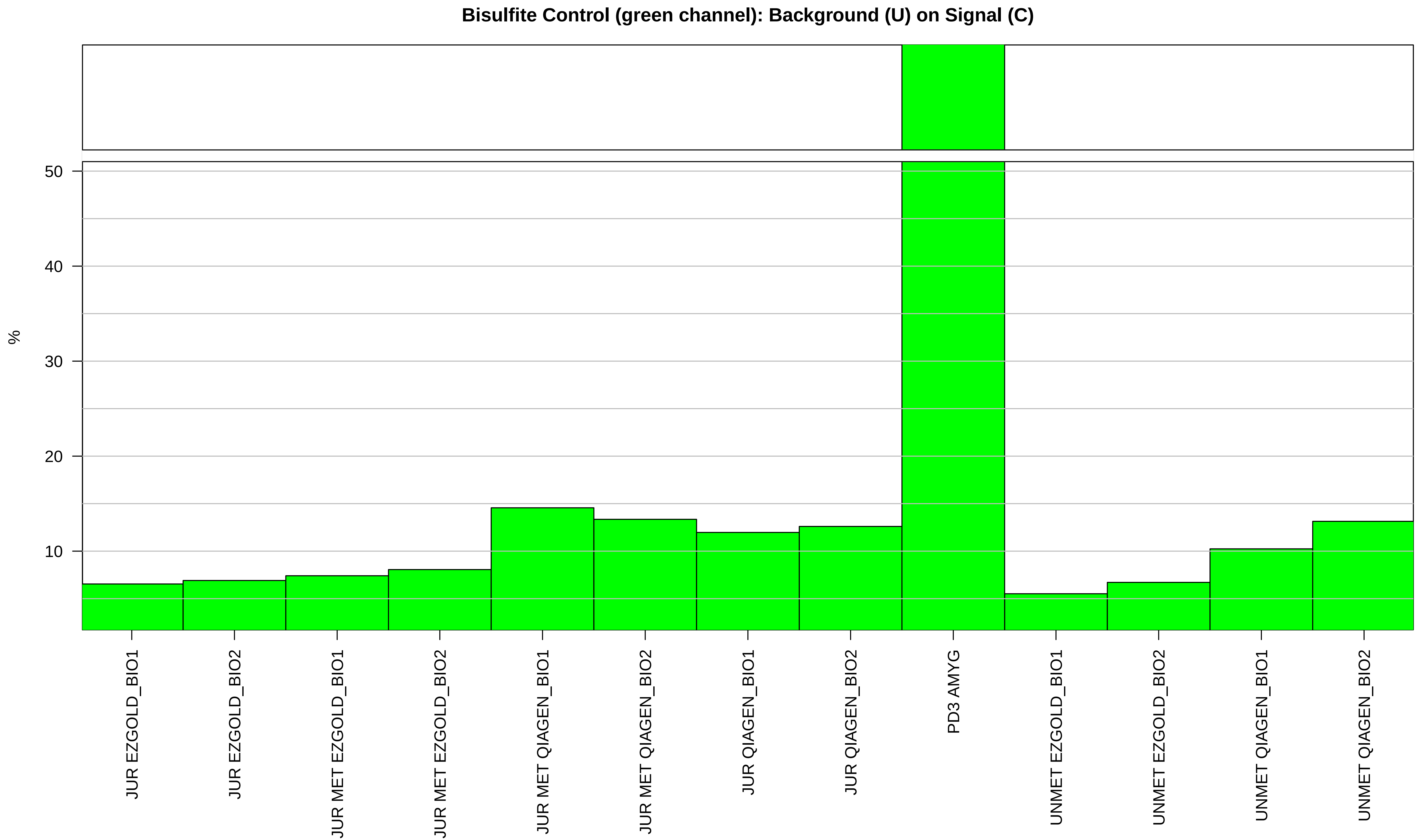

Bisulfite Control (green channel): Background (U) on Signal (C)
%
50
40
30
20
10
JUR EZGOLD_BIO1
JUR EZGOLD_BIO2
JUR MET EZGOLD_BIO1
JUR MET EZGOLD_BIO2
JUR MET QIAGEN_BIO1
JUR MET QIAGEN_BIO2
JUR QIAGEN_BIO1
JUR QIAGEN_BIO2
PD3 AMYG
UNMET EZGOLD_BIO1
UNMET EZGOLD_BIO2
UNMET QIAGEN_BIO1
UNMET QIAGEN_BIO2

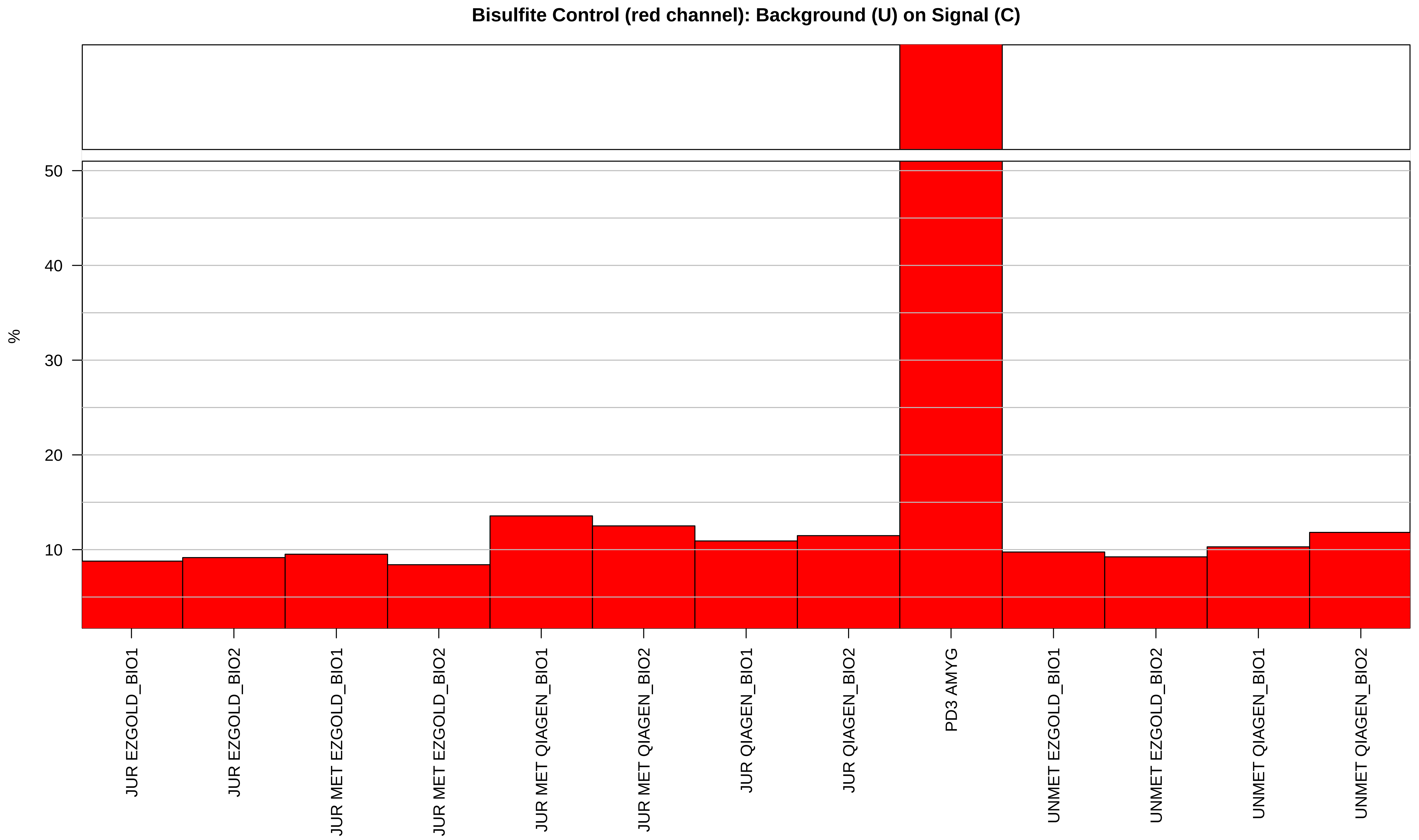

Bisulfite Control (red channel): Background (U) on Signal (C)
%
50
40
30
20
10
JUR EZGOLD_BIO1
JUR EZGOLD_BIO2
JUR MET EZGOLD_BIO1
JUR MET EZGOLD_BIO2
JUR MET QIAGEN_BIO1
JUR MET QIAGEN_BIO2
JUR QIAGEN_BIO1
JUR QIAGEN_BIO2
PD3 AMYG
UNMET EZGOLD_BIO1
UNMET EZGOLD_BIO2
UNMET QIAGEN_BIO1
UNMET QIAGEN_BIO2

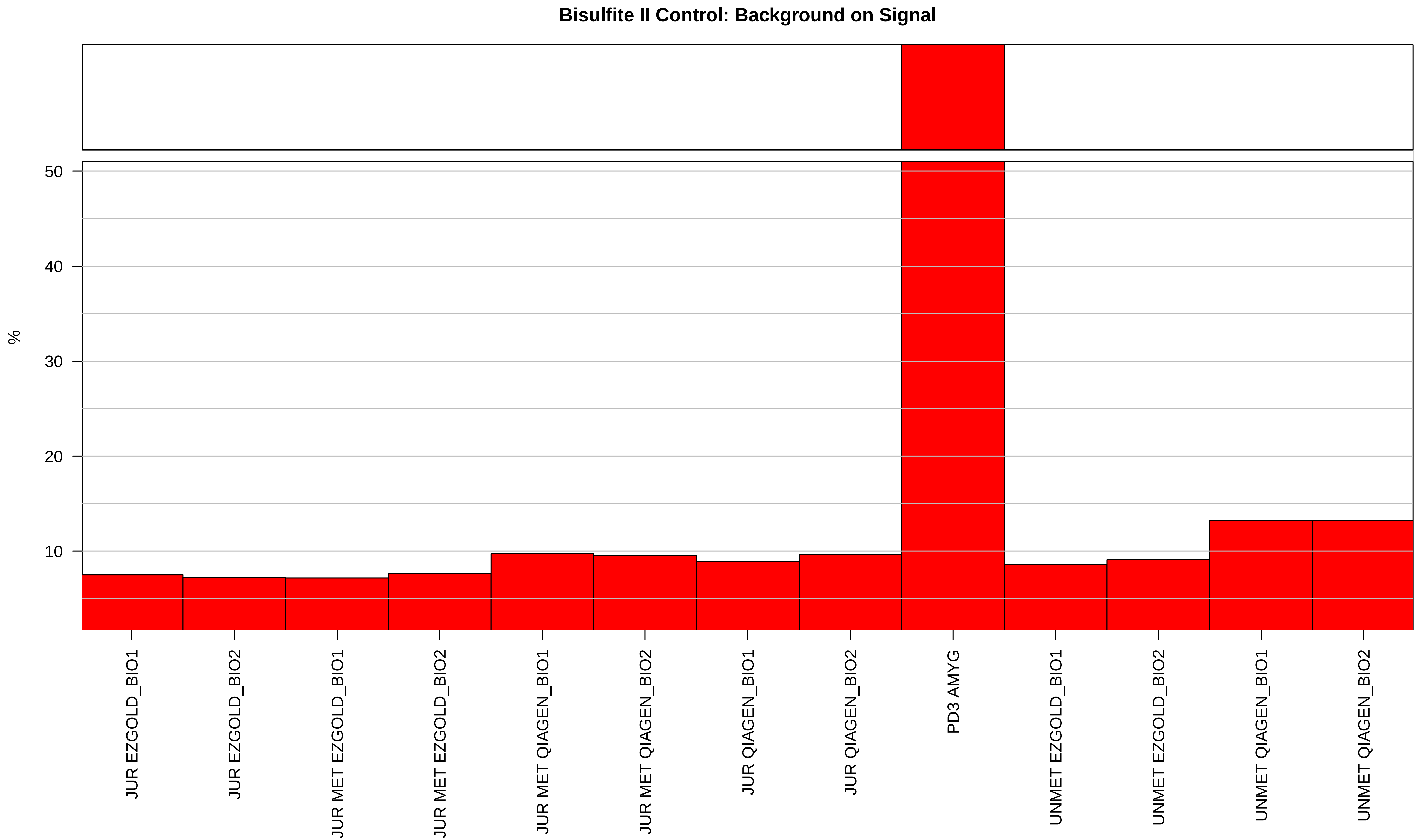
Bisulfite II Control: Background on Signal
%
50
40
30
20
10
JUR EZGOLD_BIO1
JUR EZGOLD_BIO2
JUR MET EZGOLD_BIO1
JUR MET EZGOLD_BIO2
JUR MET QIAGEN_BIO1
JUR MET QIAGEN_BIO2
JUR QIAGEN_BIO1
JUR QIAGEN_BIO2
PD3 AMYG
UNMET EZGOLD_BIO1
UNMET EZGOLD_BIO2
UNMET QIAGEN_BIO1
UNMET QIAGEN_BIO2

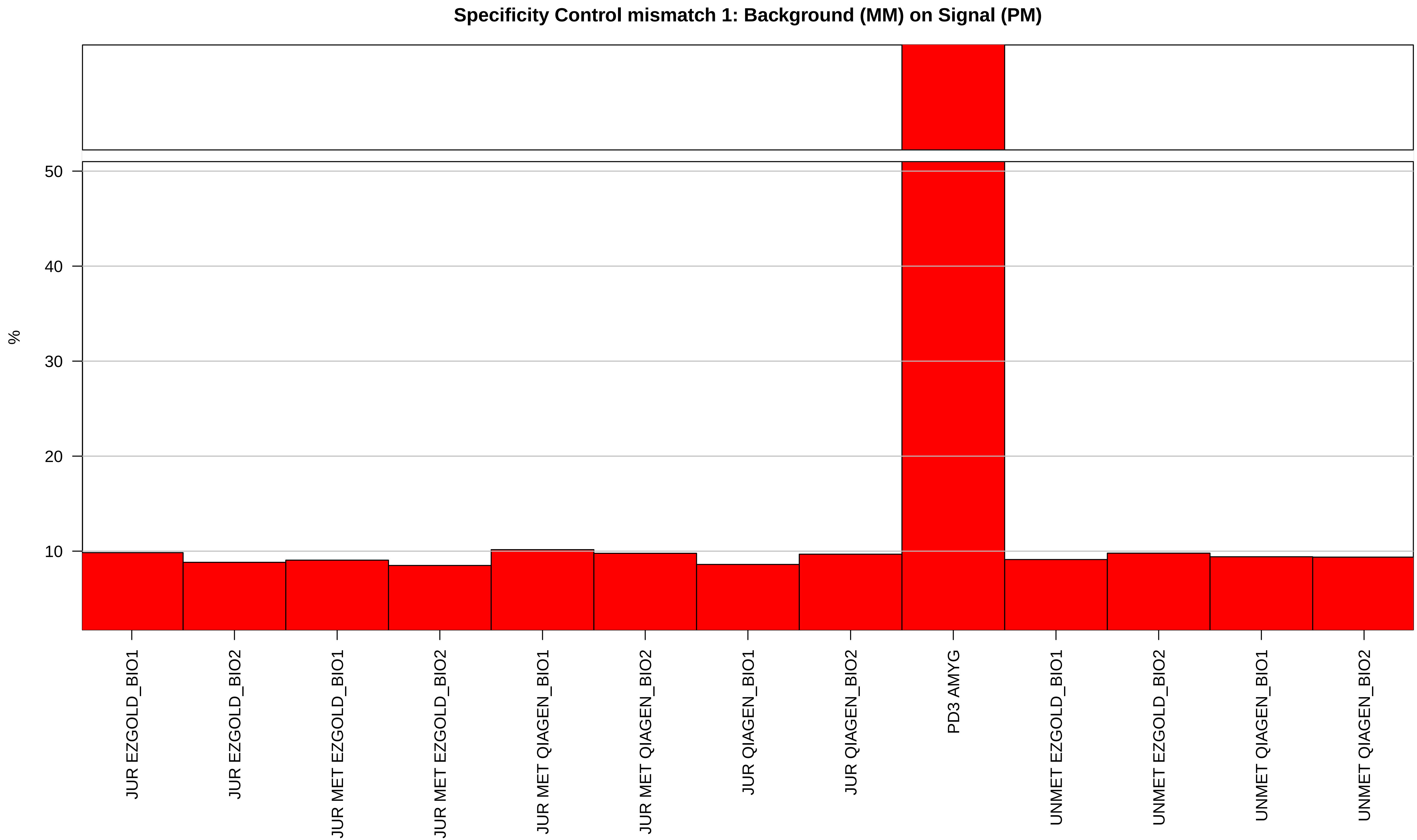

Specificity Control mismatch 1: Background (MM) on Signal (PM)
%
50
40
30
20
10
JUR EZGOLD_BIO1
JUR EZGOLD_BIO2
JUR MET EZGOLD_BIO1
JUR MET EZGOLD_BIO2
JUR MET QIAGEN_BIO1
JUR MET QIAGEN_BIO2
JUR QIAGEN_BIO1
JUR QIAGEN_BIO2
PD3 AMYG
UNMET EZGOLD_BIO1
UNMET EZGOLD_BIO2
UNMET QIAGEN_BIO1
UNMET QIAGEN_BIO2

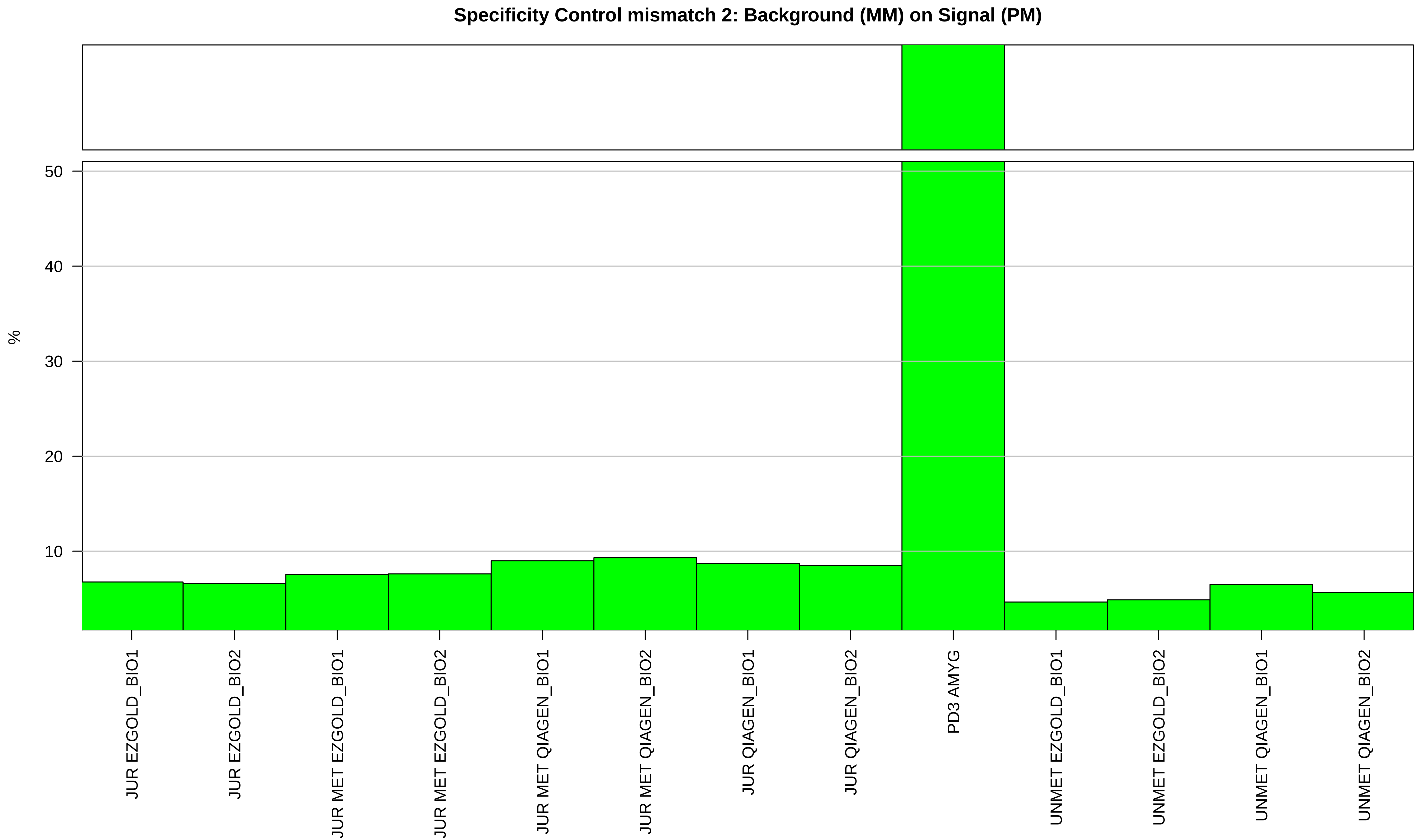

Specificity Control mismatch 2: Background (MM) on Signal (PM)
%
50
40
30
20
10
JUR EZGOLD_BIO1
JUR EZGOLD_BIO2
JUR MET EZGOLD_BIO1
JUR MET EZGOLD_BIO2
JUR MET QIAGEN_BIO1
JUR MET QIAGEN_BIO2
JUR QIAGEN_BIO1
JUR QIAGEN_BIO2
PD3 AMYG
UNMET EZGOLD_BIO1
UNMET EZGOLD_BIO2
UNMET QIAGEN_BIO1
UNMET QIAGEN_BIO2

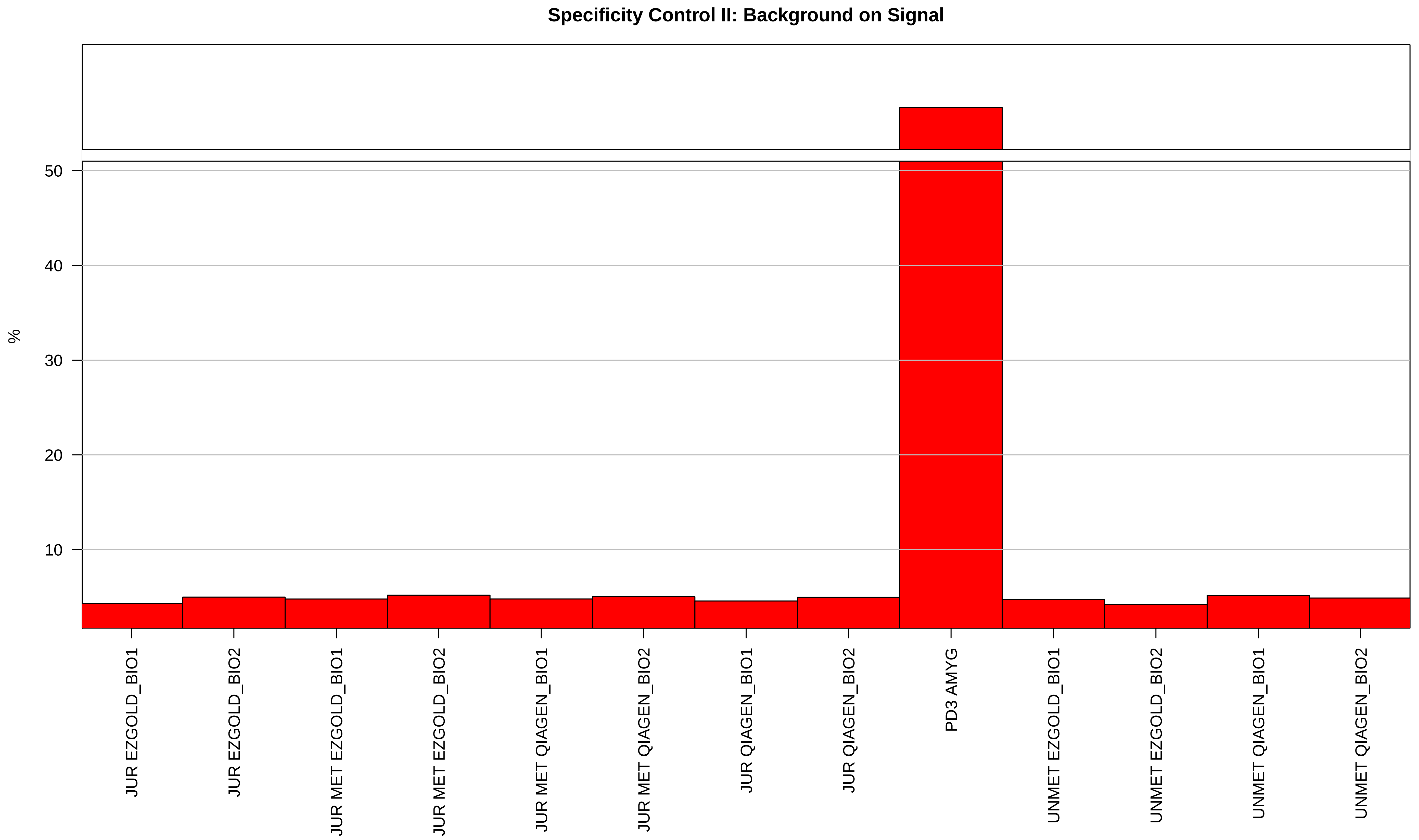
Specificity Control II: Background on Signal
%
50
40
30
20
10
JUR EZGOLD_BIO1
JUR EZGOLD_BIO2
JUR MET EZGOLD_BIO1
JUR MET EZGOLD_BIO2
JUR MET QIAGEN_BIO1
JUR MET QIAGEN_BIO2
JUR QIAGEN_BIO1
JUR QIAGEN_BIO2
PD3 AMYG
UNMET EZGOLD_BIO1
UNMET EZGOLD_BIO2
UNMET QIAGEN_BIO1
UNMET QIAGEN_BIO2

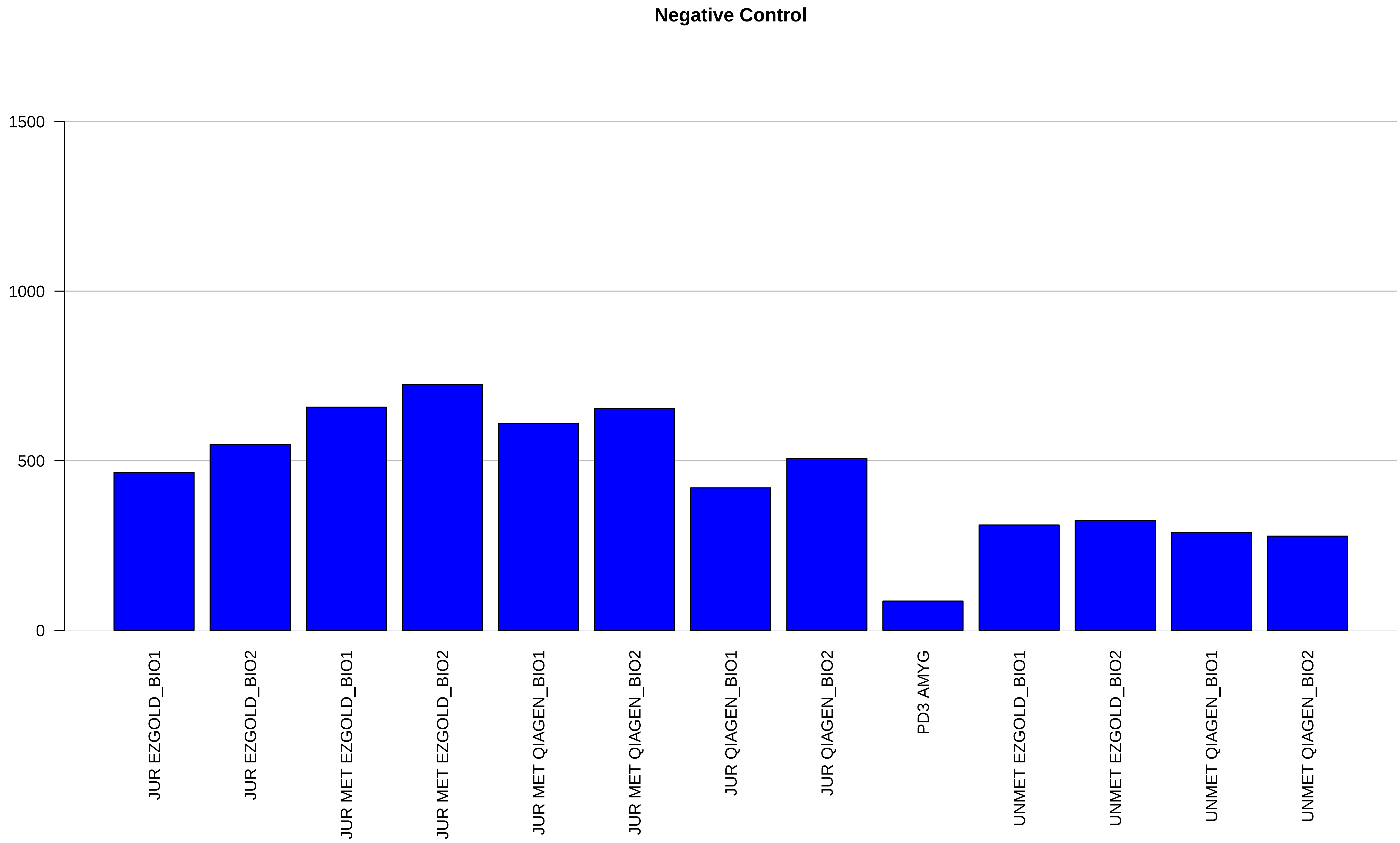

Negative Control
1500
1000
500
0
JUR EZGOLD_BIO1
JUR EZGOLD_BIO2
JUR MET EZGOLD_BIO1
JUR MET EZGOLD_BIO2
JUR MET QIAGEN_BIO1
JUR MET QIAGEN_BIO2
JUR QIAGEN_BIO1
JUR QIAGEN_BIO2
PD3 AMYG
UNMET EZGOLD_BIO1
UNMET EZGOLD_BIO2
UNMET QIAGEN_BIO1
UNMET QIAGEN_BIO2

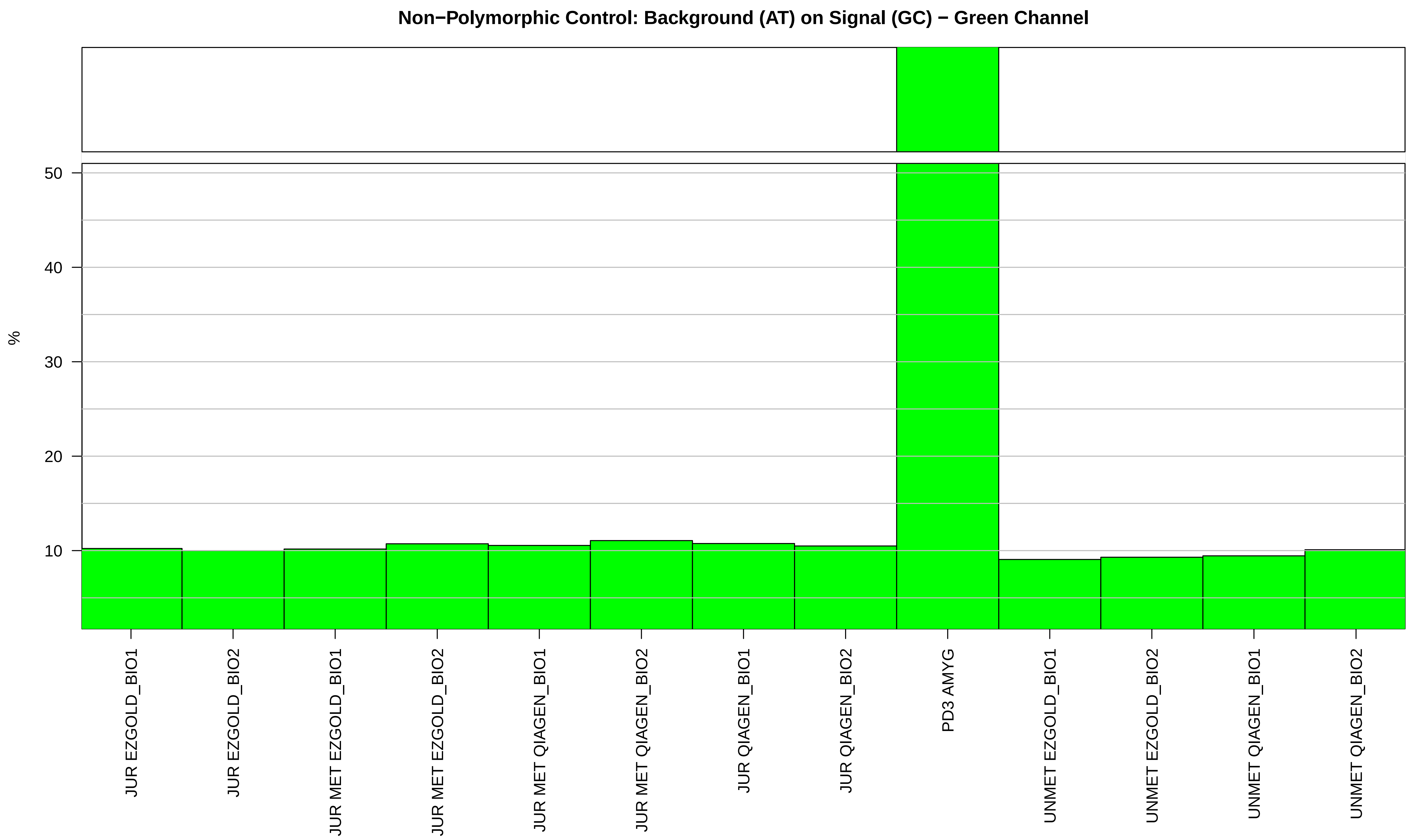

Non−Polymorphic Control: Background (AT) on Signal (GC) − Green Channel
%
50
40
30
20
10
JUR EZGOLD_BIO1
JUR EZGOLD_BIO2
JUR MET EZGOLD_BIO1
JUR MET EZGOLD_BIO2
JUR MET QIAGEN_BIO1
JUR MET QIAGEN_BIO2
JUR QIAGEN_BIO1
JUR QIAGEN_BIO2
PD3 AMYG
UNMET EZGOLD_BIO1
UNMET EZGOLD_BIO2
UNMET QIAGEN_BIO1
UNMET QIAGEN_BIO2

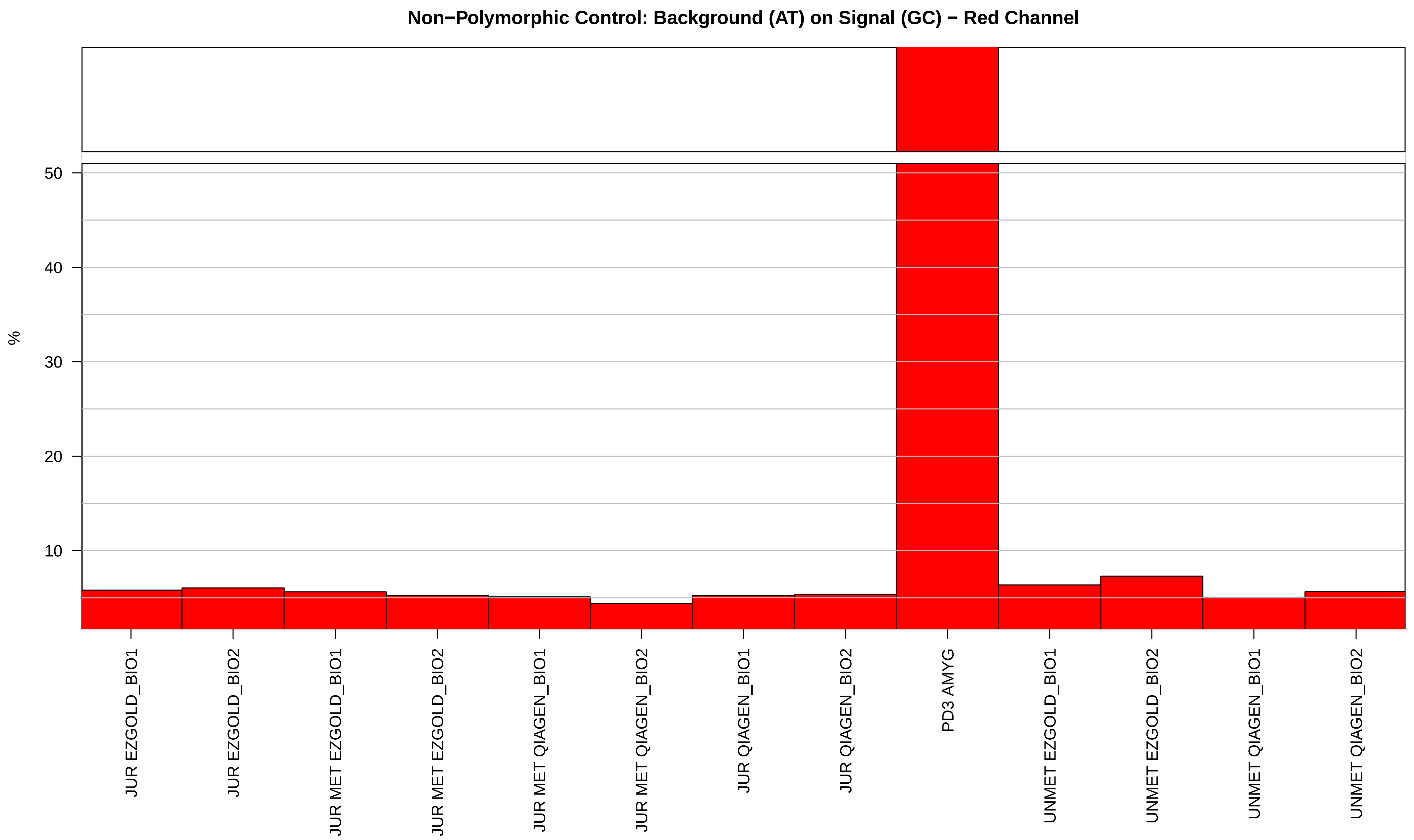

Non−Polymorphic Control: Background (AT) on Signal (GC) − Red Channel
%
50
40
30
20
10
JUR EZGOLD_BIO1
JUR EZGOLD_BIO2
JUR MET EZGOLD_BIO1
JUR MET EZGOLD_BIO2
JUR MET QIAGEN_BIO1
JUR MET QIAGEN_BIO2
JUR QIAGEN_BIO1
JUR QIAGEN_BIO2
PD3 AMYG
UNMET EZGOLD_BIO1
UNMET EZGOLD_BIO2
UNMET QIAGEN_BIO1
UNMET QIAGEN_BIO2

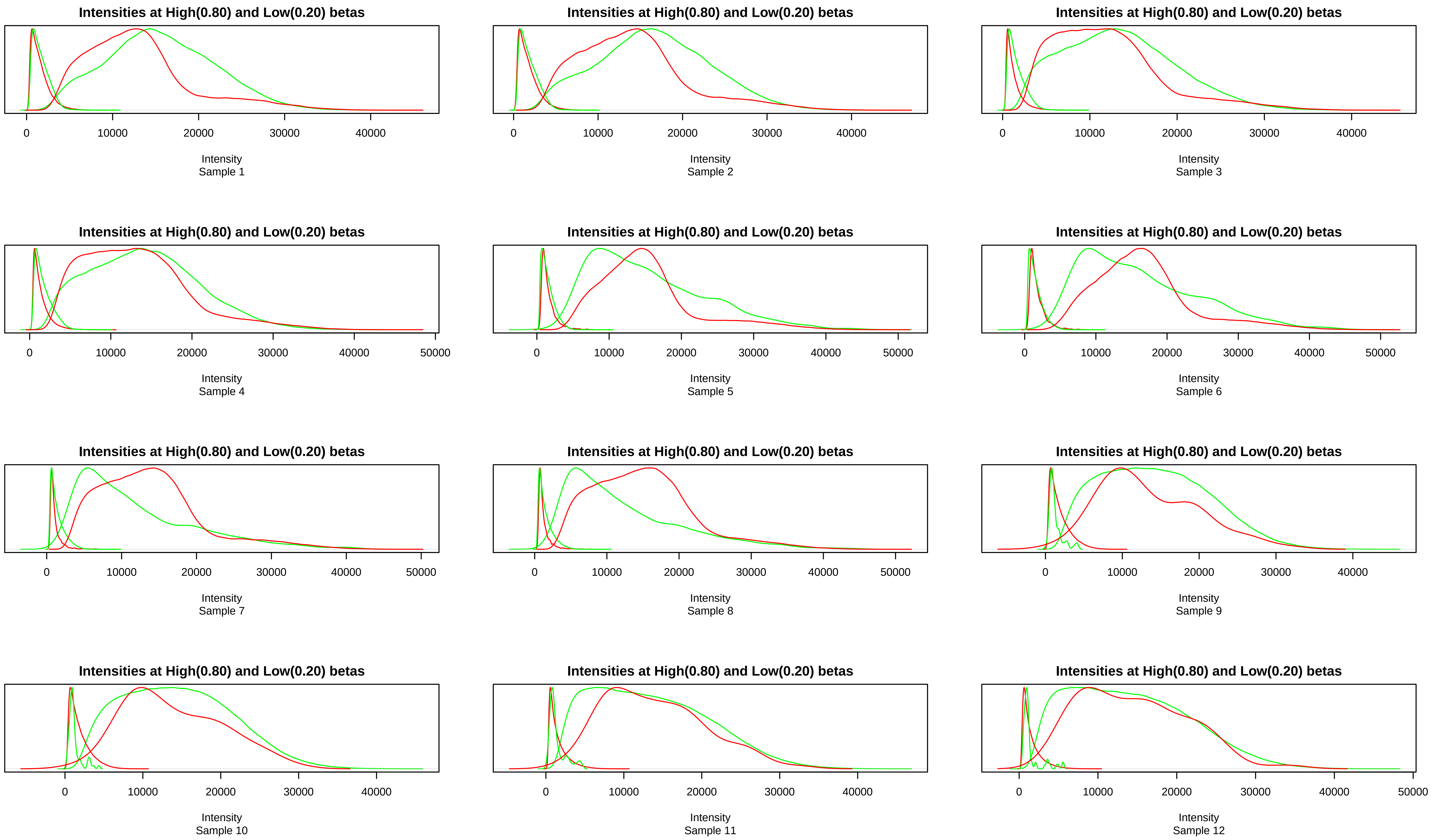
Intensities at High(0.80) and Low(0.20) betas
0
10000
20000
30000
40000
Intensity
Sample 1
Intensities at High(0.80) and Low(0.20) betas
0
10000
20000
30000
40000
Intensity
Sample 2
Intensities at High(0.80) and Low(0.20) betas
0
10000
20000
30000
40000
Intensity
Sample 3
Intensities at High(0.80) and Low(0.20) betas
0
10000
20000
30000
40000
50000
Intensity
Sample 4
Intensities at High(0.80) and Low(0.20) betas
0
10000
20000
30000
40000
50000
Intensity
Sample 5
Intensities at High(0.80) and Low(0.20) betas
0
10000
20000
30000
40000
50000
Intensity
Sample 6
Intensities at High(0.80) and Low(0.20) betas
0
10000
20000
30000
40000
50000
Intensity
Sample 7
Intensities at High(0.80) and Low(0.20) betas
0
10000
20000
30000
40000
50000
Intensity
Sample 8
Intensities at High(0.80) and Low(0.20) betas
0
10000
20000
30000
40000
Intensity
Sample 9
Intensities at High(0.80) and Low(0.20) betas
0
10000
20000
30000
40000
Intensity
Sample 10
Intensities at High(0.80) and Low(0.20) betas
0
10000
20000
30000
40000
Intensity
Sample 11
Intensities at High(0.80) and Low(0.20) betas
0
10000
20000
30000
40000
50000
Intensity
Sample 12

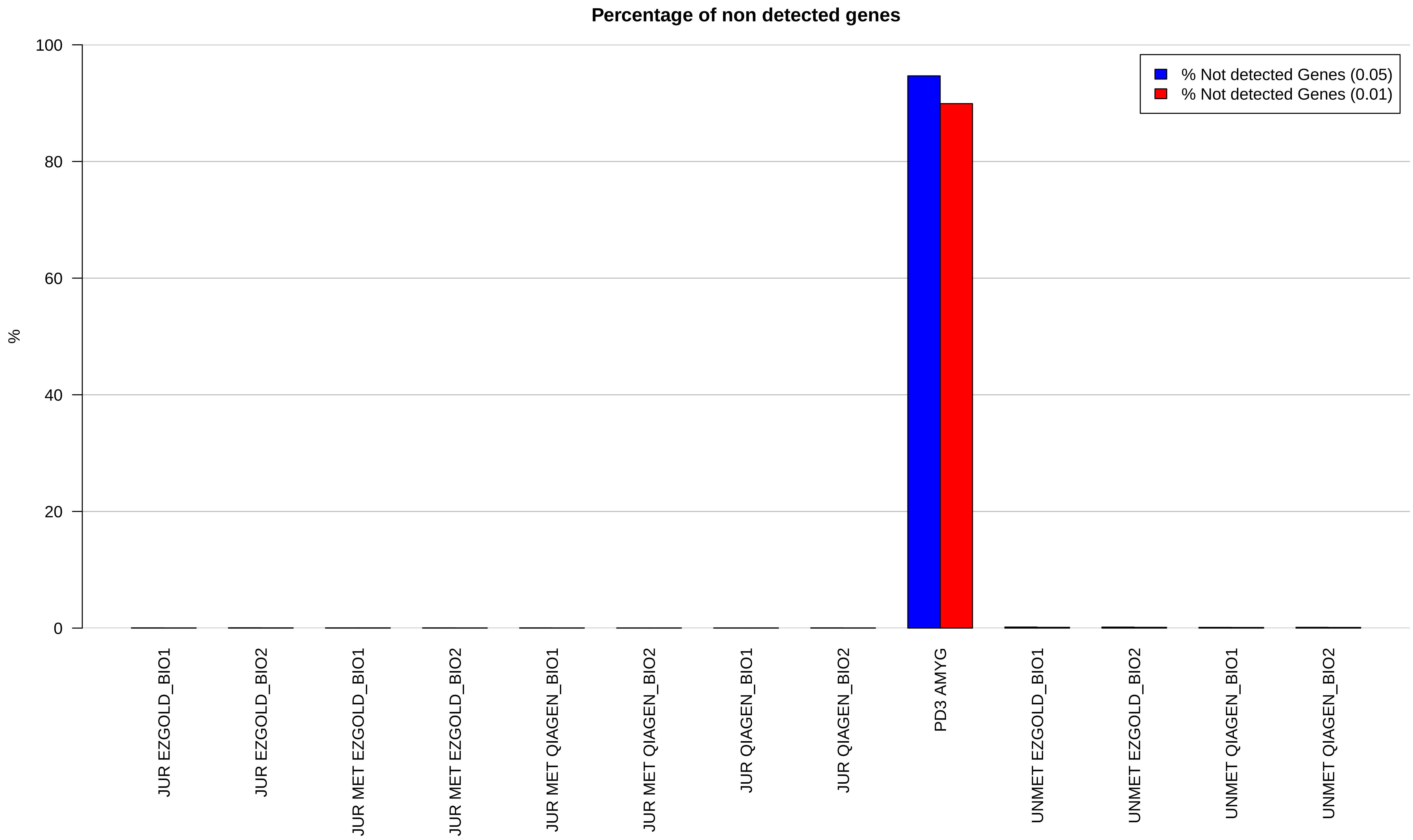

Percentage of non detected genes
100
80
60
40
20
0
%
% Not detected Genes (0.05)
% Not detected Genes (0.01)
JUR EZGOLD_BIO1
JUR EZGOLD_BIO2
JUR MET EZGOLD_BIO1
JUR MET EZGOLD_BIO2
JUR MET QIAGEN_BIO1
JUR MET QIAGEN_BIO2
JUR QIAGEN_BIO1
JUR QIAGEN_BIO2
PD3 AMYG
UNMET EZGOLD_BIO1
UNMET EZGOLD_BIO2
UNMET QIAGEN_BIO1
UNMET QIAGEN_BIO2

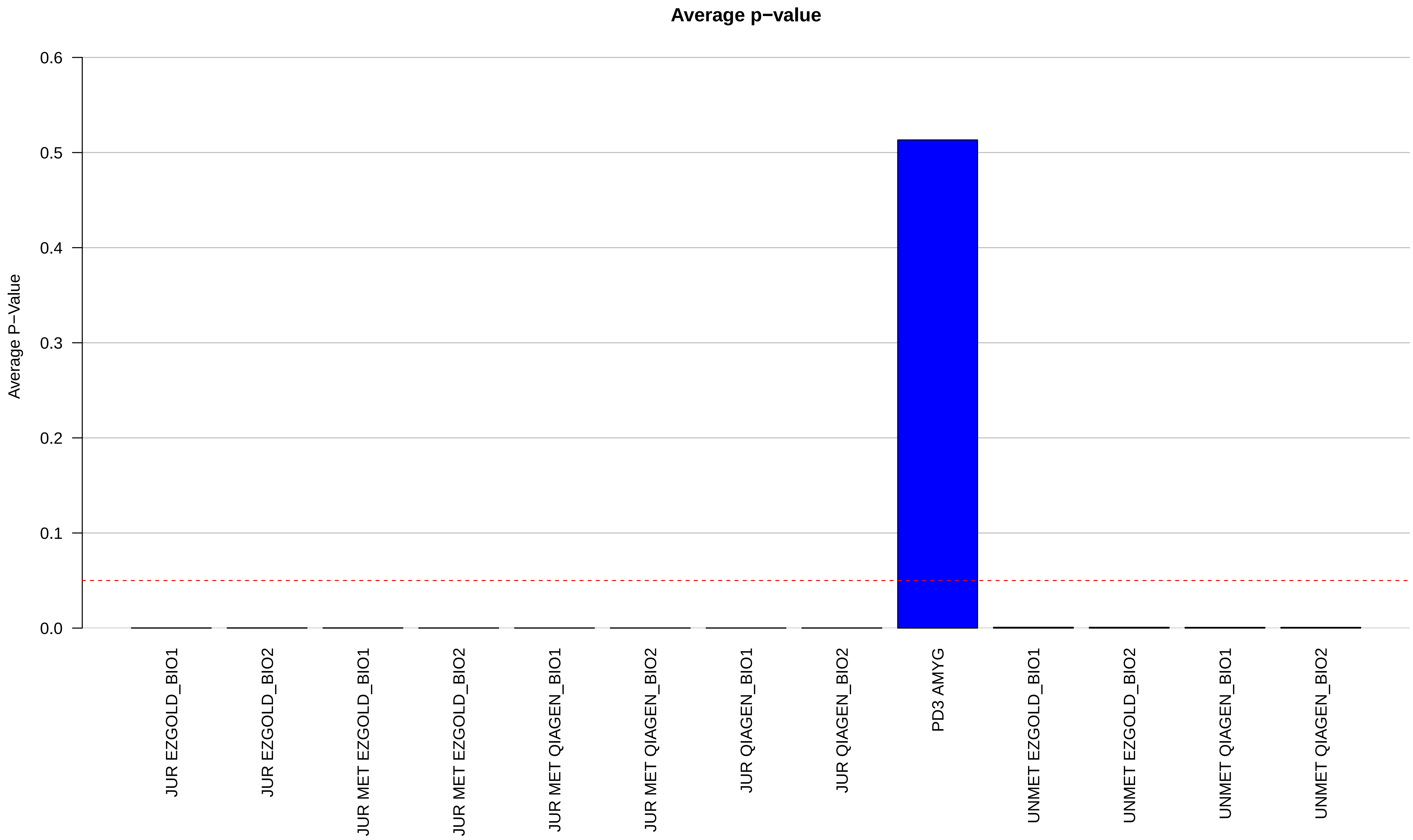
Average p−value
Average P−Value
0.0
0.1
0.2
0.3
0.4
0.5
0.6
JUR EZGOLD_BIO1
JUR EZGOLD_BIO2
JUR MET EZGOLD_BIO1
JUR MET EZGOLD_BIO2
JUR MET QIAGEN_BIO1
JUR MET QIAGEN_BIO2
JUR QIAGEN_BIO1
JUR QIAGEN_BIO2
PD3 AMYG
UNMET EZGOLD_BIO1
UNMET EZGOLD_BIO2
UNMET QIAGEN_BIO1
UNMET QIAGEN_BIO2

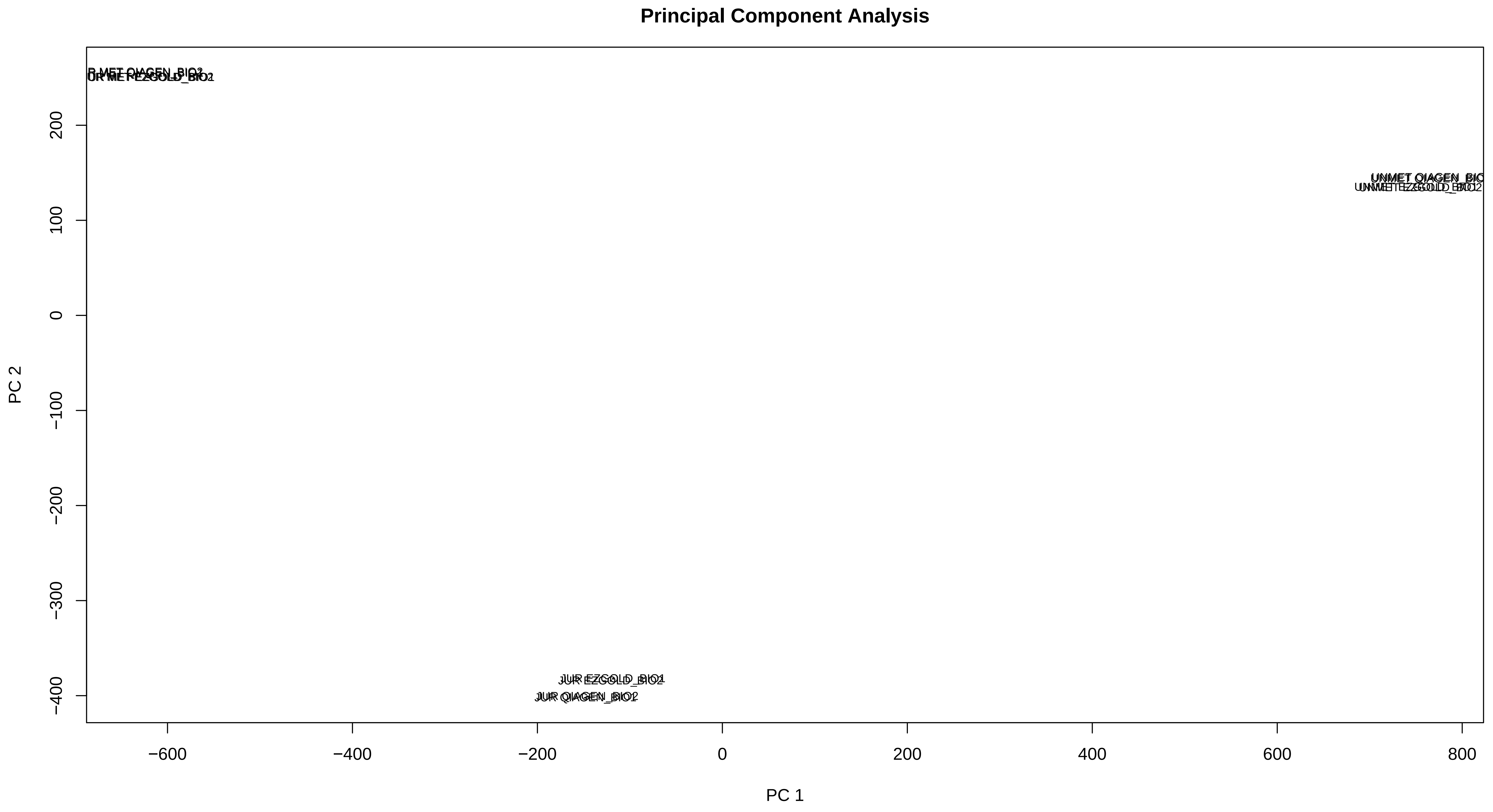

Principal Component Analysis
PC 1
PC 2
−600
−400
−200
0
200
400
600
800
−400
−300
−200
−100
0
100
200
UNMET QIAGEN_BIO
JUR EZGOLD_BIO1
JUR EZGOLD_BIO2
JUR QIAGEN_BIO2
JUR QIAGEN_BIO1

Hierarchical Clustering

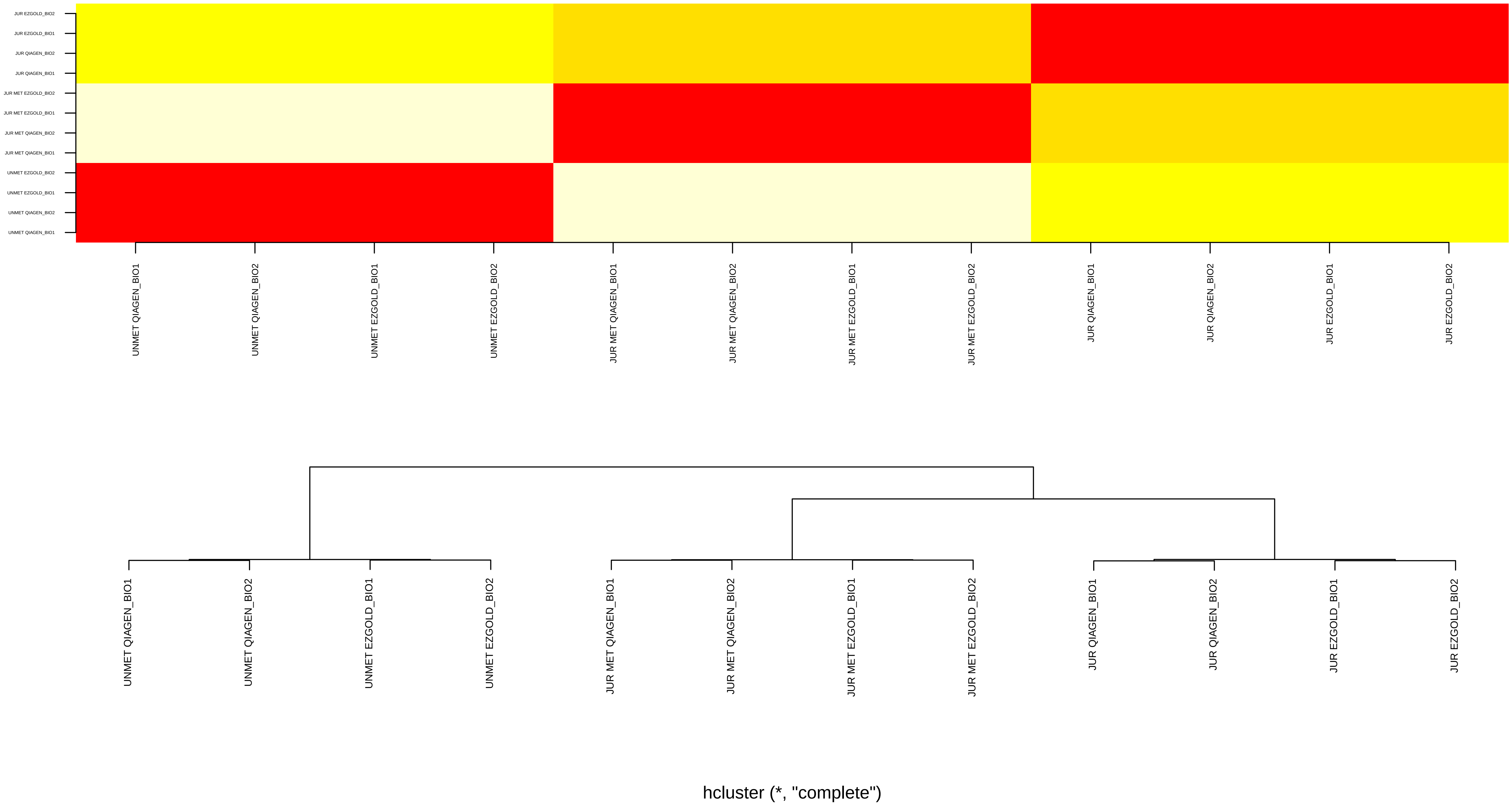

Supplement: Additional file 1 — Full QC report PDF file containing all the figures produced by HumMeth27QCReport during the QC analysis. [file 1756-0500-4-546-S1.PDF]
